# Antimicrobials in Orthopedic Infections: Overview of Clinical Perspective and Microbial Resistance

**DOI:** 10.3390/medicina60121988

**Published:** 2024-12-02

**Authors:** Bogdan Huzum, Ana Clara Aprotosoaie, Ovidiu Alexa, Paul Dan Sîrbu, Bogdan Puha, Bogdan Veliceasa, Riana Maria Huzum

**Affiliations:** 1 Department of Orthopaedic and Traumatology, Faculty of Medicine “Grigore T. Popa”, University of Medicine and Pharmacy of Iasi, 700115 Iasi, Romania; bogdan.huzum@umfiasi.ro (B.H.); ovidiu.alexa@umfiasi.ro (O.A.); paul.sirbu@umfiasi.ro (P.D.S.); bogdan.puha@umfiasi.ro (B.P.); bogdan.veliceasa@umfiasi.ro (B.V.); 2 Faculty of Pharmacy “Grigore T. Popa”, University of Medicine and Pharmacy of Iasi, 700115 Iasi, Romania; 3 Department of Radiology, Faculty of Medicine “Grigore T. Popa”, University of Medicine and Pharmacy of Iasi, 700115 Iasi, Romania; riana-maria.huzum@umfiasi.ro

**Keywords:** orthopedic infections, antibiotics, microbial resistance

## Abstract

Orthopedic infections are challenging pathologies that impose a heavy burden on patients and the healthcare system. Antimicrobial therapy is a critical component of the successful management of orthopedic infections, but its effectiveness depends on patient-, surgery-, drug-, and hospital-related factors. The dramatic increase in the emergence of multidrug-resistant microbial strains necessitates new clinical approaches in order to prevent or limit this phenomenon and to ensure a favorable therapeutic outcome. The present paper reviews the currently available antimicrobial strategies in the management of orthopedic infections, highlighting their clinical use related to the occurrence of microbial resistance. Some approaches for reducing antibiotic resistance emergence in orthopedics are also presented. The use of antibiotics tailored to the microorganism’s sensitivity profile, patient factors, and pharmacokinetic profile in terms of monotherapy or combinations, the understanding of microbial pathogenicity and resistance patterns, strict control measures in healthcare facilities, the development of new antimicrobial therapies (drugs, devices, technologies), and patient education for improving compliance and tolerance are some of the most important tools for overcoming microbial resistance.

## 1. Introduction

Orthopedic infections are a major human health issue with a significant psychosocial and financial burden. They are associated with chronic pain, physical disability, and prolonged morbidity, and they are considered an important cause of mortality. Orthopedic infections are costly, often implying surgical interventions, long-term antimicrobial treatment, and prolonged hospitalization. Infectious postoperative complications almost double the cost of treatment for orthopedic trauma patients. Previous studies reported a median cost of over USD 108,000 for an infected orthopedic patient (initial hospitalization and readmission) in the US [1]. The combined annual hospital costs related to periprosthetic joint infections (PJIs) of the hip and knee were estimated to be USD 1.85 billion by 2030 in the US [2]. In Europe, the annual financial burden of PJIs is appreciated to be over USD 35,000 per patient [2].

The incidence of orthopedic-trauma-related infections ranges from 5% to 10% depending on the location and severity of the injury [3]. Surgical site infections (SSIs) in orthopedic surgery are one of the most serious and challenging postoperative complications, especially when implants are involved. The rate of infection incidence varies from 1.3% to 10% in hip and knee procedures and from 12% to 25% in foot and ankle surgery [4]. PJIs can affect approximately 2–3% of patients with hip and knee arthroplasty [5] or 1–2.4% of joint replacement subjects [6]. The prevalence of PJIs associated with resistant and atypical pathogens is increasing, surpassing 50% of infections in some cases [5]. Postoperative sepsis is a serious surgical complication involving any type of infection that can lead to sepsis, severe sepsis, or septic shock, and it is associated with high morbidity and mortality. It is estimated that postoperative sepsis occurs in more than 1% of elective procedures and in more than 4% of non-elective procedures [7]. The incidence of postoperative septicemia in orthopedic trauma patients is reported to be about 1.6%, and it is greater than the rate of sepsis in nontraumatic orthopedic patients (0.5%) [8].

A number of factors have been identified to increase the risk for SSIs and PJIs and to modulate the outcome of anti-infective therapy. They include patient-, surgery-, and hospital-related items. Patient-related factors mainly involve age, gender, body mass index, morbid obesity, malnutrition, hypoalbuminemia, excessive smoking and alcohol consumption, severe immunodeficiency, diabetes mellitus, connective tissue diseases (rheumatoid arthritis), chronic corticosteroid therapy, chronic liver and renal disorders, prolonged wound drainage, history of previous surgery, and primary diagnosis and socioeconomic profile [5,9]. Surgery-related factors include preoperative skin preparation, prolonged intervention time, bilateral procedures, allogeneic blood transfusion, deep venous thrombosis prophylaxis and coagulopathy, and failure of antibiotic prophylaxis. Prolonged hospitalization, admission from a healthcare facility, and surgeon volume of procedures also constitute risk factors for orthopedic-device-related infections [9]. Significant risk factors for both postoperative sepsis and septic shock include male gender, diabetes, chronic obstructive pulmonary disease, hypertension, dependent functional status, American Society of Anesthesiology Score ASA ≥ 3, and corticosteroid use. Preoperative ventilator dependence and congestive heart failure are unique risk factors for septic shock [10]. The most important biomarkers used for the evaluation of infections and other postoperative complications are presented in Table S1 (Supplementary Material) [11,12,13,14,15,16,17].

In an attempt to provide valuable prognostic tools for the likelihood of SSIs development, the Centers for Disease Control and Prevention (US) designed a surgical wound classification system (SWC) based on the degree of microbial contamination. The SWC includes four classes (I, clean; II, clean/contaminated; III, contaminated; IV, dirty) with scores of SSIs risk of 1 to 5%, 3 to 11%, 10 to 17%, and more than 27%, respectively. However, the current data show that SWC has a questionable predictive power for SSIs in orthopedic patients [18,19]. Onyekwelu et al. (2017) [20] reviewed 400 orthopedic surgery cases covering a wide spectrum of interventions (long bones, shoulders, knees, ankles, wrists, pelvis), and they did not show a correlation between SSIs incidence and SWC grade. Only certain variables such as diabetes mellitus and lower extremity injury proved to be positive prognostic factors for SSIs. To more accurately predict SSIs in orthopedic patients, future risk-stratifying models should categorize wounds based on injury severity, site, and patient comorbidities. It is necessary to consider the inclusion of intrinsic risk factors such as poor soft-tissue coverage and the need for soft-tissue flaps and vascular repair in wound classification systems for orthopedic patients [18,19,20].

The therapy of orthopedic infections is difficult and challenging, and it requires a pharmacotherapeutic approach and, sometimes, surgical procedures. The phenomenon of multidrug-resistant organisms emergence makes successful antimicrobial treatment even more challenging.

The emergence and rapid global spread of drug-resistant microorganisms, mainly multi- and pan-resistant bacteria (so-called superbugs), are a serious public health threat worldwide. Antimicrobial-resistant infections (AMR) are considered the third leading cause of death after cardiovascular diseases [21]. The Global Antimicrobial Resistance and Use Surveillance System (GLASS) estimated that 1.27 million deaths globally were attributable to AMR in 2019, and nearly 5 million deaths were indirectly associated with AMR according to a study from 2022 [21,22]. Multidrug-resistant pathogens were reported in 37.5–65.5% of postoperative orthopedic infections [23].

This paper discusses the main antimicrobial agents currently used in orthopedic infections, highlighting their clinical applicability and the issue of microbial resistance. It also presents clinical approaches that can reduce microbial bioburden, such as the decolonization of surgical patients with antistaphylococcal agents, as well as strategies for prophylaxis related to antimicrobial resistance in orthopedics.

## 2. Microbiota in the Orthopedic Infections

Gram-positive bacteria are responsible for the majority of orthopedic infections, accounting for 60–80% of the etiological pathogens [24,25]. *Staphylococcus aureus* and coagulase-negative staphylococci (CoNS) are the most common pathogens [26]. Other Gram-positive organisms including streptococci, enterococci, *Corynebacteria* and anaerobes may be involved in specific conditions [24,27]. The local microenvironment, the presence and diversity of implants, the physical and chemical properties of biomaterials, the anatomical sites of surgery, individual tissue responses, and microorganisms’ adaptation all influence the spectrum and distribution of pathogens [28,29]. *S aureus* is the most prevalent and most harmful single causative bacterium, accounting for about 40% of the PJIs [30]. It exhibits remarkable virulence, especially in patients with comorbidities, and can be involved in both early-onset and delayed- or late-onset infections [29]. Additionally, *S. aureus* is recognized for its high levels of antibiotic resistance and its ability to form biofilms on foreign materials, which enhances the severity of infections. Besides the biofilm-forming properties, it is able to cause a direct infection of bone cells by the invasion of the osteocyte lacuno-canalicular network [26]. Factors such as older age, male gender, obesity, diabetes mellitus, immunosuppression, ethnicity, recent hospitalization and misuse of antibiotic treatments increase *S. aureus* colonization [30]. Strains of *S. aureus* isolated from orthopedic implant-associated infections include MRSA and vancomycin-resistant *S. aureus* (VRSA), but they are also frequently resistant to ciprofloxacin and aminoglycosides (e.g., amikacin, gentamicin, netilmicin, and tobramycin) [31]. The World Health Organization (WHO) recommends preoperative screening and the eradication of both MRSA and methicillin-sensitive *S. aureus* (MSSA) in orthopedic surgery [32,33]. Nowadays, CoNS such as *Staphylococcus epidermidis*, *S. lugdunensis*, *S. schleiferi*, *S. caprae*, *S. mitis* and *S. hominis* represent a concerning bacterial population with an increasing role in PJIs. The growing incidence of antibiotic-resistance in CoNS makes them even more refractory to antimicrobial treatments. CoNS are robust biofilm-producing pathogens and have a high capacity to acquire antibiotic resistance genes supporting plurivalent protection from infection clearance. Moreover, CoNS can enhance the antimicrobial resistance of other pathogens, both CoNS and non-CoNS [34]. Male sex, open injury, trauma, and lower extremity are considered the significant risk factors for CoNS infections [35]. *Streptococcus* spp. are the second most common bacteria isolated from implant-associated infections, with *S. agalactiae* being the major species. They specifically colonize the vascular network within bones, but cause less osteolysis than *Staphylococcus aureus* [26].

*Enterococcus* species are found in 3–15% of skeletal infections, with the highest rates identified in vertebral infections [5,26]. *Enterococcus faecalis* is the most frequently isolated species, and high levels of antibiotic resistance have been observed in both *E. faecalis* and *E. faecium* [5]. The percentage of vancomycin resistance increased from 9% in 2014 to 17% in 2020 for *Enterococcus faecium* [36]. *Cutibacterium acnes* is the most commonly encountered anaerobic Gram-positive bacillus [26]. It is especially associated with shoulder arthroplasty and rotator cuff surgery, but other joint prostheses, such as hip and knee prostheses, may also be notably infected. Although *C. acnes* is susceptible to many antibiotics commonly used in orthopedic infections (beta-lactams, quinolones, and rifampicin), it is showing increasing resistance to clindamycin [37].

Gram-negative bacteria are also detected in both monomicrobial and polymicrobial orthopedic infections. They mainly include *Escherichia coli*, *Pseudomonas aeruginosa*, *Klebsiella pneumoniae*, *Proteus*, *Pasteurella*, and *Serratia* species. Some studies report their involvement in 4–7% of PJIs, 30% of SSIs after hip interventions, and 10% of SSIs after knee procedures [38]. However, an increase in PJIs due to Gram-negative bacteria (23–44%) has been reported recently [25]. Among Gram-negative bacteria, multidrug-resistant *Acinetobacter*, extended spectrum β-lactamase producing Enterobacteriaceae (ESBLs), and multidrug-resistant *Pseudomonas aeruginosa* have been reported in orthopedic implant-associated infections. Increasing resistance to carbapenems raises significant concerns regarding the successful outcome of orthopedic interventions. In most cases of *Acinetobacter* infections, carbapenemresistance is combined with resistance to fluoroquinolones and aminoglycosides [39]. The percentage of *Klebsiella pneumoniae* resistant to carbapenems increased from 8% in 2014 to 10% in 2020, and the rate of resistance to ciprofloxacin varied from 8.4% to 92.9% for *Escherichia coli* and from 4.1% to 79.4% for *Klebsiella pneumoniae* [36,40]. As recently noticed by the WHO, carbapenem-resistant enterobacteria, *Pseudomonas*, and *Acinetobacter* are critical targets in the development of new antimicrobial strategies and antibiotics [39,40].

## 3. Antibiotics and Antimicrobial Chemotherapeutics

The choice of antimicrobial treatment should be tailored according to the causal pathogens, as revealed by culture results of isolated organisms, their sensitivity profile and patient risk factors, such as comorbidities, allergies, previous debridements, previous antibiotic therapies, the presence of prosthetic material, vascular supply to the affected site, individual drug tolerance, and antibiotic penetration into bone. Recent guidelines recommend selective nasal and skin decolonization for patients at high risk of being MRSA carriers before surgery [41]. The issues of decolonization, antimicrobial prophylaxis, and treatment in orthopedic infections are discussed below.

### 3.1. Bacterial Colonization and Decolonization

Colonization with *S. aureus* is an important modifiable risk factor for SSIs after orthopedic surgery [42]. Carriers of *S. aureus* have a 9–10 times greater incidence of SSIs than non-carriers and MRSA colonization leads to a fourfold higher risk of PJIs [30]. The nasal cavity and skin are the relevant sites of *S. aureus* colonization. The prevalence of MSSA nasal carriage ranges from 15% to 36.9% in the general population, while MRSA colonization occurs in 0.6–7% of individuals [30]. Age, gender, obesity, diabetes mellitus, immunosuppression, ethnicity, recent hospitalization and misuse of antibiotics are considered risk factors for *S. aureus* colonization [30]. Preoperative *S. aureus* screening and decolonization in orthopedic surgery have been recommended by the WHO since 2016 [43]. Numerous subsequent studies have demonstrated a significant decrease (even over 60%) in the prevalence of SSIs following the implementation of screening and a decolonization protocol. A meta-analysis conducted in 2020, including seven retrospective studies and two prospective studies on primary knee and hip arthroplasties, showed a significant reduction in the risk of *S. aureus* SSIs in patients in the screening and decolonisation group compared to the control group (OR, 0.43, 95%CI 0.31–0.59) [44]. A recent meta-analysis of 12 independent studies, including 56,930 cases, conducted by Bianco Prevot et al. (2024) [42], also indicated the effectiveness of *S. aureus* decolonization in patients undergoing knee or hip prosthesis implantation, reducing total PJIs and those caused by MSSA and MRSA. Nasal screening is the most reliable for detecting *S. aureus* carriage, being superior to sampling from the throat, axilla, or perineum. However, testing multiple sites improves the detection rate. Young et al. (2017) showed that the use of both nasal and throat swabbing increased the total detection rate by 10% [45]. Testing is performed using bacterial culture-based techniques and real-time polymerase chain reaction (RT-PCR), with RT-PCR being a more sensitive and faster method [30].

The most commonly used protocol for the effective decolonization of *S. aureus* includes the intranasal application of topical mupirocin 2% twice daily, accompanied by or without daily showers or chlorhexidine skin wipes for 5 days prior to surgery [30,42]. If the 5-day period of mupirocin application is not completed before surgery, the treatment should be completed postoperatively [46].

Mupirocin is highly effective in eradicating intranasal MRSA colonization in orthopedic patients. Additionally, it is moderately active against Gram-negative bacteria. The antibiotic disrupts bacterial RNA and protein synthesis by inhibiting isoleucyl-transfer RNA synthetase [47]. The development of mupirocin resistance has been reported in 6.6% of European patients, according to recent data [43].Clorhexidine body wash is used as a supplement to mupirocin to reduce the skin bacterial load [30]. Several alternative decolonization strategies (intranasal alcohol-based antiseptics, intranasal povidone-iodine, tea tree essential oil, lysostaphin, and omiganan pentahydrochloride, phototherapy, or probiotics use) have been suggested, but their clinical applicability needs to be explored in larger and more robust trials [46].

### 3.2. Prophylaxis

Regardless of the type of orthopedic surgery, antibiotic prophylaxis is a key strategy in preventing surgical site infections. Furthermore, dual antibiotic prophylaxis may be indicated for high-risk surgical patients and in conditions with high MRSA rates.

Numerous professional organizations, including the American Academy of Orthopedic Surgeons (AAOS), the Centers for Disease Control and Prevention (CDC), the European Centre for Disease Prevention and Control (ECDC), and the WHO, advocate for antibiotic prophylaxis in orthopedic surgery. Their recommendations are updated based on new data and the discovery of antibiotic resistance patterns. According to some studies, antibiotic prophylaxis can reduce the absolute risk of infections by 81% in patients undergoing total hip and knee replacement [48,49]. Additionally, prophylactic antibiotics can decrease the rate of SSIs following arthroplasty from 4–8% to 1–3% [50].

The timing of administration, the type of antibiotics, and the duration of prophylaxis are crucial in preventing SSIs. ECDC recommends that perioperative antibiotic prophylaxis (PAP) be administered within 60 min before the incision, except for vancomycin and fluoroquinolones, where the optimum time is at the moment of anesthetic induction [50]. A single dose is preferred, with subsequent doses administered depending on the duration of the procedure, the pharmacokinetics of the antibiotic, and major blood loss during surgery. The discontinuation of PAP is indicated within 24 h after initiation of surgery [50]. However, extended prophylaxis may be necessary for obese, diabetic, or immunocompromised patients [51]. The AAOS guidelines specify the administration of prophylactic antibiotics one hour before the incision or two hours for vancomycin and fluoroquinolones; the duration of prophylaxis is typically no longer than 24 h [51]. Failure to administer antibiotic prophylaxis within the recommended 2-h window is associated with a 2-to 6-fold increase in the rates of SSIs [27]. To achieve effective prophylaxis, the antibiotic should be active against a broad spectrum of pathogens, reach serum concentrations above the minimum inhibitory concentration (MIC), and have a sufficiently long half-life to maintain adequate levels throughout the procedure [27]. The optimal prophylaxis would theoretically include a combination of glycopeptides with aminoglycosides, or glycopeptides with carbapenems, piperacillin-tazobactam, or similar broad-spectrum agents that are effective against both Gram-positive and Gram-negative pathogens to prevent new postoperative superinfections [52]. The most commonly used prophylactic agents are cefazolin, clindamycin, vancomycin, gentamicin, cefuroxime, and ciprofloxacin [51].

### 3.3. Antimicrobial Treatment

Post-interventional antimicrobial treatment is administered based on the susceptibility of identified pathogens, including intravenous agents in the initial stage [5]. Prosthetic infections require antibiotic treatment for a duration of 6 weeks to 6 months, with 6 weeks being the most common time duration—this typically includes 2 weeks of parenteral therapy followed by 4 weeks of oral therapy [53,54]. The definitive antibiotic therapy for chronic osteomyelitis is determined based on the bone biopsy culture and should be administered for a minimum of 6 weeks, including parenteral agents. However, some authors recommend 6 to 8 weeks of intravenous treatment, followed by 3 or more months of oral administration to prevent therapy failure and recurrence of infection. Effective antimicrobial treatment for chronic osteomyelitis requires good drug penetration into bone tissue and a long-term duration of treatment [55]. In fact, the bone and joint tissue penetration profile of antibiotics is a critical factor in determining a successful clinical outcome. Drug characteristics (protein binding, size, lipophilicity, degree of ionization) and tissue properties (capillary fenestration, inflammation, bone variety) significantly influence the concentration of antibiotics at the target site. The synovial fluid appears to be more accessible than bone. Based on the pharmacokinetic/pharmacodynamic (PK/PD) index of antibiotic and the epidemiological cutoff value (ECOFF) for targeted pathogens, cefazolin, cefuroxime, linezolid, flucloxacillin, vancomycin, and ciprofloxacin provide adequate average exposure with standard dosing regimens [56]. In implant-associated infections, antibiotics must target both stationary-phase bacteria and biofilm aggregates [57]. Aside from the systemic therapy, the local delivery of antimicrobials can be an important adjunctive treatment strategy. This is particularly beneficial for managing dead space in fracture-related infections [58]. The peculiar characteristics of local antimicrobials include broad-spectrum activity, adequate release from the carrier, low rates of resistance and hypersensitivity, and a reduced risk of toxicity. Gentamicin, tobramycin, vancomycin, and clindamycin are the most clinically used antibiotics for local therapy in orthopedic infections [59]. These antibiotics are incorporated into bone cement, collagen, and other bone void fillers (such as calcium sulfate) for clinical use. In addition to commercially available preparations, local antibiotics are also incorporated into off-label carriers such as polymethyl methacrylate (PMMA), biodegradable ceramics and hydrogels [59,60]. Only heat-stable antibiotics, such as aminoglycosides, glycopeptides, tetracyclines, and quinolones can be incorporated into PMMA. Vancomycin, tobramycin, and gentamicin are the most commonly used [60]. Antibiotics-infused PMMA systems are primarily used for the prophylaxis of open fractures with bone or soft tissue loss, or for the treatment of osteomyelitis and fracture-related infections [61]. Low-dose antibiotics in bone cement are ideal for prophylaxis, being defined as ≤ 1 g of antibiotic per 40 g of PMMA powder [61]. The effective release and sustained therapeutic concentrations of antibiotics in the treatment of orthopedic infections require high-dose antibiotic-loaded bone cement, defined as ≥ 3.6 g of antibiotic per 40 g of PMMA powder [61]. Biodegradable ceramics, made from calcium sulfate or phosphate, have an improved release profile and maintain antibiotic concentration longer than PMMA [60]. Hydrogels are newer antibiotic carriers, capable of including various antibiotics, and are mainly used for prophylaxis with targeted effects [61].

Improving antibiotic activity and bioavailability through antimicrobial combinations and system delivery, along with a better understanding of the mechanisms behind orthopedic infections and microbial pathogenicity, could provide significant strategies to overcome these complex infections and to decrease the risk of microbial resistance [53]. The quality of prosthesis/implant removal, two-stage surgery, and antibiotic washing are also valuable factors that can enhance antimicrobial effectiveness. In this context, the clinical applicability (combinations, targeted administration based on the anatomy and microbiology of the infection) of the most important classes of antimicrobials in orthopedic infections are discussed below, considering the issue of microbial resistance. The main antimicrobial drugs used in the treatment of orthopedic infections are listed in Table S2 (Supplementary Material) [62,63,64].

#### 3.3.1. Beta-Lactam Antibiotics

They include penicillins, cephalosporins, carbapenemes, and monobactams.

Both natural penicillins (penicillin G, penicillin V) and narrow-spectrum penicillins (nafcillin, oxacillin, dicloxacillin) are most active on Gram-positive bacteria, while broad-spectrum penicillins (ampicillin, amoxicillin) are effective against both Gram-positive and Gram-negative pathogens [65].

Cephalosporins are divided into five generations. The first generation (cefazolin, cephalexin) is predominantly active against Gram-positive bacteria, particularly *Staphylococcus* spp. and *Streptococcus* spp. [38,66]. The third-generation cephalosporins (ceftriaxone, cefixime) are less active against Gram-positive bacteria than the second generation (cefuroxime), but they offer extended coverage of Gram-negative bacteria, including *Enterobacteriaceae*, *Neisseria* spp., and *Haemophylus influenzae* [66]. Fourth-generation (cefepime) and fifth-generation cephalosporins (ceftaroline, ceftobiprole) are effective against multi-resistant organisms, with the latter being particularly active against MRSA and penicillin-resistant pneumococci [66].

Among beta-lactams, carbapenems (imipenem, meropenem, doripenem) are potent antibiotics with broad-spectrum activity against Gram-negative bacteria (*Pseudomonas aeruginosa* and most Enterobacteriaceae, including strains that produce AmpC beta-lactamase and extended-spectrum beta-lactamase-ESBL), anaerobes (e.g., *Bacteroides* spp.), and Gram-positive cocci (methicillin-sensitive staphylococci and streptococci) [67,68]. The combination of carbapenems with aminoglycosides acts synergistically against *P. aeruginosa* [63]. The antibacterial activity of beta-lactams is related to the inhibition of cell wall biosynthesis, disrupting cell morphology and eventually causing cell lysis and death. They target the peptidoglycan layer (PGN) of the bacteria cell wall, blocking the formation of PGN peptide cross-linkages by inhibiting transpeptidases, the enzymes responsible for PGN synthesis [69]. Due to their broad-spectrum effect on microbial strains commonly involved in orthopedic infections, good safety profile, long half-life, and good penetration in bone, synovium, and muscle, cephalosporins are by far the most popular choice of antibiotics forprophylaxis in orthopedic and trauma surgery, including total joint arthroplasty [4,49]. According to the American Society of Health System Pharmacists (ASHP), cefazolin is the most used antibiotic in preoperative prophylaxis, with the combination of cefazolin and gentamicin being the second most common regimen, while third-generation cephalosporins are the third most widely used antibiotics [49]. Third-generation cephalosporins have been strongly linked to the development of *Clostridium difficile*, and a single dose of cephalosporin prophylaxis is sufficient to promote *Clostridium* difficile colonization [27]. The trend in Western literature is to use second-generation cephalosporins (cefuroxime) as prophylactic antibiotics, administered 30 min to 1 h before skin incision, and preferably continued as an intravenous infusion for 24 h to 3 days postoperatively. Cefuroxime shows high tissue and serum bioavailability after a single dose, and it is effective in preventing perioperative infections [49,70]. For patients allergic to penicillin/cephalosporins, the use of vancomycin, teicoplanin, or clindamycin as prophylactic antibiotics is recommended [38]. The standard antibiotic therapy for MSSA infections includes penicillinase-resistant penicillins (nafcillin/oxacillin/flucloxacillin), with the first-generation cephalosporin cefazolin as an alternative [38].

Flucloxacillin exerts a good activity against *Staphylococcus aureus* (but it is ineffective against MRSA and 90% of CoNS), and it is routinely used as a first-line anti-staphylococcal treatment for orthopedic infections [27]. However, the joint tissue penetration of flucloxacillin may be affected by its high protein binding rate [71]. The combination with rifampicin can increase flucloxacillin levels by 44.5% [58]. Parenteral beta-lactams (oxacillin, nafcillin, cefazolin) are suitable agents for the initial treatment of chronic staphylococcal osteomyelitis [55]. In contrast, the emergence of beta-lactam resistance does not typically occur during antistaphylococcal therapy [72]. According to the Infectious Diseases Society of America guidelines for PJIs, beta-lactam antibiotics are the agents of choice for streptococcal infections. Intravenous therapy (penicillin or ceftriaxone) should be given for up to a week, followed by oral therapy with amoxicillin [53,58]. In cases of penicillin allergy, trimethoprim/sulfamethoxazole, doxycycline, and clindamycin are acceptable alternative options [58].

Co-amoxiclav, a combination of amoxicillin and clavulanic acid, a beta-lactamase inhibitor, is the recommended first-line antibiotic for open fractures according to the guidelines of the British Orthopaedic Association and the British Association of Plastic, Reconstructive, and Aesthetic Surgeons. Both components show excellent penetration into synovial fluid, with concentrations almost equivalent to those in serum [27,71]. In the fracture-related infections (FRI) caused by enterococci (especially *Enterococcus faecalis*) that are sensitive to ampicillin or amoxicillin, these antibiotics should be the initial choice, with up to a week of intravenous therapy followed by oral amoxicillin [58]. Although the bactericidal action of beta-lactams is considered inferior to that of fluoroquinolones, beta-lactam antibiotics such as piperacillin/tazobactam, cefepime, ceftazidime, or a carbapenem (except ertapenem) should be used as the initial treatment for FRI caused by Enterobacteriaceae (*Escherichia coli*, *Klebsiella* spp., *Enterobacter* spp.) [58].

In patients with Gram-negative infections who meet the criteria for debridement and implant retention, intravenous therapy with cephalosporins or carbapenems, followed by oral quinolones, is recommended [73].

The Gram-positive anaerobe *Cutibacterium acnes* should initially be treated with benzylpenicillin or ceftriaxone, followed by oral rifampicin in combination with amoxicillin, doxycycline, or quinolones [58].

For Gram-negative anaerobes, therapy with intravenous ampicillin/sulbactam or amoxicillin/clavulanic acid, followed by oral metronidazole, is recommended [58].

For patients with grade III fractures, a first-generation cephalosporin should be given in conjunction with an aminoglycoside [74]. Additionally, the combination of ceftazidime and ciprofloxacin has been used in patients with *Pseudomonas aeruginosa*-infected osteosynthetic material without implant removal [53].

#### 3.3.2. Rifampicin (Rifampin)

Rifampicin, also known as rifampin (US, Canada), is a semisynthetic derivative of rifamycin, a macrocyclic antibiotic produced by the actinomyte *Amycolatopsis rifamycinica* (previously known as *Streptomyces mediterranei* sin. *Nocardia mediterranei* and *Amycolatopsis mediterranei)* [75]. Rifampicin is highly active against most Gram-positive bacteria but exerts more limited activity against Gram-negative pathogens due to its poor ability to penetrate the bacterial cell membrane [76]. The antibiotic shows potent activity against *Staphylococcus aureus* and can inhibit adhering stationary-phase organisms, making it particularly effective in bone infections. It is especially useful as an anti-biofilm agent in patients with staphylococcal orthopedic implant-associated infections undergoing debridement and implant retention (DAIR), as well as in patients with chronic osteomyelitis [24,26,77]. Rifampicin can penetrate the staphylococcal biofilm and exert bactericidal activity; in addition, rifampicin can prevent biofilm formation [78]. However, monotherapy with rifampicin leads to the rapid emergence of resistance [58], so it must always be used in combination with other antimicrobial agents [26]. Combination therapy is more effective in preventing the emergence of resistant pathogens and the failure of prosthetic joint infection treatment [53]. Antimicrobial agents with high oral bioavailability and good bone penetration, such as quinolones, trimethoprim-sulfamethoxazole (high dose, 960 mg every 8 h), doxycycline, or clindamycin, are highly recommended as partners in rifampicin combinations [77]. Rifampicin combination therapy should be reserved for patients with DAIR, and the following conditions are necessary for this therapy: fair skin and soft tissue condition (dry wounds), a stable implant, susceptibility to the antibiotic, low bacterial load after debridement surgery, and initial antimicrobial intravenous treatment [79,80]. Adjunctive rifampicin therapy in orthopedic-device-related infections is typically started with 450 mg twice daily or 300 mg twice daily in cases of intolerance [81]. Fluoroquinolones are the first choice as partners in rifampicin combination therapy [81]. The advantages of this association include excellent bioavailability, good intracellular penetration, and antistaphylococcal activity [53]. The moxifloxacin–rifampicin and levofloxacin–rifampicin combinations have proven to be successful in patients with early acute PJIs caused by MSSA [58]. Also, the rifampicin and levofloxacin combination is effective as an anti-biofilm agent in patients with staphylococcal infections and chronic osteomyelitis, preventing the emergence of resistance [55]. This same combination of antibiotics has also shown good efficacy in treating *Cutibacterium acnes* infections of fractures [53]. Rifampicin is commonly very active against most streptococci, including nutritionally variant species. The use of rifampicin combination therapy has been reported in few case reports involving PJIs caused by beta-hemolytic streptococci [81]. The addition of rifampicin to antibiotics such as ciprofloxacin, linezolid, daptomycin, or tigecyline has been successful in the treatment of acute enterococcal PJIs, either with debridement or two-stage exchange arthroplasty [5]. These combinations are also effective in reducing *Enterococcus faecalis* biofilms, and rifampicin combined with daptomycin or tigecycline acts synergistically in invasive enterococcal infections [81]. Some patients are unable to tolerate rifampicin due to side effects or drug interactions. Severe nausea is a common issue, especially in elderly patients [80].

Hepatic failure has been reported with the use of fusidic acid and rifampicin combinations in MRSA infections [24]. Fusidic acid is a second-line antimicrobials often combined with rifampicin. It reaches high intracellular concentrations and has potent activity against *Staphylococcus aureus* [24]. However, the coadministration of these agents results in low plasma levels of fusidic acid, likely due to the induction of the liver enzyme CYP3A4 by rifampicin. This interaction can lead to the development of resistance, and a new dosing regimen for fusidic acid may be necessary [82].

#### 3.3.3. Clindamycin

Clindamycin is a semisynthetic chlorinated derivative of lincomycin from the lincosamide antibiotics group. It provides coverage against Gram-positive bacteria (*Staphylococcus* spp., *Streptococcus* spp.) and anaerobic Gram-negative organisms (*Fusobacterium*, *Prevotella*, *Bacteroides* spp.), but it is ineffective against aerobic Gram-negative bacteria and MRSA. Also, the coagulase-negative staphylococci are often resistant [83,84]. Clindamycin is widely recommended for the treatment of bone and joint infections due to its good bone and synovial fluid penetration, as well as its sustained activity against biofilm formation and bacterial adherence [24,85]. Furthermore, clindamycin has excellent oral bioavailability, making it a suitable choice for switch therapy in patients who no longer require hospital admission [24]. It has also been shown to be effective as a prophylaxis for Grade I and II open fractures [27]. Due to the bacteriostatic effect of clindamycin, the use of antibiotic combinations plays an important role in the success of treatment, particularly in the prevention of emerging microbial resistance. The addition of rifampicin to clindamycin significantly increases its antibacterial activity against both coagulase-negative and coagulase-positive staphylococci [85]. In patients with erythromycin-resistant, lincosamide-susceptible *Staphylococcus* isolates, clindamycin combination therapy should be administered only if resistance to other oral antibiotics is present or if there is known intolerance, based on bacteriological results [85]. The use of clindamycin is limited in elderly patients due to the concerns about the association with pseudomembranous colitis [24].

#### 3.3.4. Glycopeptides

Vancomycin and teicoplanin are the most used glycopeptides in clinical practice. These are natural glycosylated non-ribosomal peptides produced by various soil actinomycetes [86,87]. Their spectrum of antibacterial activity is confined to Gram-positive organisms, such as *Staphylococcus aureus*, *S. epidermidis* (mainly against methicillin-resistant strains), *Streptococcus pneumoniae*, *S. viridans*, *Enterococcus faecalis*, *E. faecium* and *Clostridium difficile*. They can also inhibit *Bacillus* spp. and *Corynebacterium* [88,89]. Compared to vancomycin, teicoplanin is more potent against certain isolates of the *Staphylococcus*, *Streptococcus,* and *Enterococcus* bacteria [86].

Vancomycin or teicoplanin are recommended as perioperative prophylactic antibiotics for patients who are MRSA colonizers undergoing total joint arthroplasty (TJA). Vancomycin is commonly used for patients known to be colonized with MRSA, and it may also be indicated in FRI with ampicillin-resistant enterococci, as well as in patients with true type I IgE-mediated reactions to the penicillin [27,38].

In addition to systemic administration, vancomycin can be locally applied. It may be added to PMMA bone cement for prophylaxis in arthroplasty, or used in antibiotic spacers or beads to prevent infection in open fractures [27]. Topical vancomycin powder has significantly decreased infection rates in spine surgery [59]. However, the frequent use of vancomycin can increase the potential for the emergence of microbial resistance, particularly vancomycin-resistant *Enterococcoccus* VRE, vancomycin-resistant *Staphylococcus aureus,* and *Clostridium difficile*, which can exhibit reduced susceptibility to vancomycin [38]. Even in vancomycin-susceptible MRSA populations, subpopulations that are vancomycin-resistant (VRSA) or vancomycin-intermediate (VISA) (heteroresistant strains) may exist, decreasing the antibiotic’s effectiveness [55].

The lower susceptibility of *S. aureus* to vancomycin may be partially attributed to staphylococcal biofilms, which promote resistance by facilitating horizontal gene transfer [90].

Due to its limited coverage of Gram-negative bacteria, vancomycin should not be used as a monotherapy. It is recommended to combine vancomycin with an aminoglycoside to ensure the adequate coverage of Gram-negative pathogens [38]. However, caution is needed when using this combination, especially with gentamicin and vancomycin, due to the increased risk of acute kidney injury [38]. Even without the combination, careful monitoring for nephrotoxicity is essential, with therapeutic serum levels of vancomycin between 15 and 20 μg/L [58]. For this reason, vancomycin dosing should be at 15 mg/kg body weight [58].

To avoid “red man syndrome”, an infusion-related hypersensitivity reaction, vancomycin should be administered intravenously at a maximum rate of 1 g per hour [38]. The timing of administration is also important for vancomicyn effectiveness. The rate of PJIs is significantly lower when the antibiotic infusion starts 45 min before the surgical incision (in hip and knee total joint arthroplasty), compared to administration closer to the start of the procedure (less than 45 min) [38].

Due to the potential issues associated with vancomycin use, including adverse reactions like marrow suppression, ototoxicity, nephrotoxicity, and also slow infusion requirements and bacterial resistance, some authors have suggested adding teicoplanin to antibiotic prophylaxis regimens that include cephalosporins [41,90]. Dual prophylaxis with teicoplanin and cefuroxime has been used in patients undergoing primary hip or knee arthroplasty and surgery for femoral neck fracture, reducing Gram-positive bacteria infection rates, particularly for *S. aureus* [41,91]. In a follow-up study, Capdevila et al. 2016 [92] found that the infection rate remained low, at 2%, one year after the implementation of dual antibiotic prophylaxis with teicoplanin and cefuroxime in patients with femoral neck fractures.

Also, adding teicoplanin (800 mg) to the usual dose of cefazolin (2 g) has been associated with a 60% reduction in PJIs rates before primary hip and knee arthroplasty [41]. Teicoplanin has a better safety profile than vancomycin, being less nephrotoxic, and can be infused over 20 min with a very low risk of “red man syndrome” [38]. It can be administered by bolus injection once daily or less frequently, allowing the continuation of parenteral treatment for patients discharged from the hospital [24]. New lipoglycopeptides, such as telavancin, oritavancin, and dalbavancin, have also been developed. They exhibit potent activity against Gram-positive bacteria (including MRSA, and to some extent VISA, streptococci, and clostridia), and are similar to or more effective than vancomycin. The remarkably long half-lives of oritavancin and dalbavancin allow for infrequent dosing (single dose of 1200 mg for oritavancin and 1000 mg on day 1 followed by 500 mg on day 8 for dalbavancin), which could be beneficial for outpatient therapy in the future [55,93]. Although currently approved only for the treatment of acute bacterial skin and soft tissue infections, other potential indications, including orthopedic infections, have been examined in some case reports. Thus, telavancin and dalbavancin have been successfully used in the treatment of acute and chronic osteomyelitis in small series of patients [55]. Compared to vancomycin, televancin has a lower potential for developing microbial resistance. However, it is also highly nephrotoxic, necessitating weekly kidney function monitoring [62].

#### 3.3.5. Aminoglycosides

Aminoglycosides are potent bactericidal antibiotics that are particularly active against aerobic Gram-negative bacteria, such as *Escherichia coli*, *Klebsiella pneumoniae*, *Enterobacter cloacae, E. aerogenes*, *Proteus* spp., *Morganella* spp., and *Serratia* spp. They are also effective against *Staphylococcus aureus* (including MRSA, vancomycin-intermediate and -resistant isolates), *Pseudomonas aeruginosa* and, to a lesser extent, *Acinetobacter baumanii* [94].

Aminoglycosides remain an important class of antibiotics, especially when the organisms of interest are resistant to other drugs, such as β-lactams and fluoroquinolones.

The most commonly used aminoglycosides are gentamicin, tobramycin, and amikacin [95]; gentamicin and tobramycin are the main aminoglycoside antibiotics used in prosthetic surgery [96]. While gentamicin and tobramycin generally display comparable antimicrobial properties, tobramycin is particularly potent against *Pseudomonas aeruginosa* and has the lowest resistance rate. Amikacin is the preferred choice for treating infections caused by ESBL-producing strains. Also, both amikacin and tobramycin are highly effective against *Acinetobacter baumannii* infections [95].

Despite their hydrophilic nature, aminoglycosides can penetrate bone and joint tissues. In synovial fluid, both amikacin and gentamicin can achieve concentrations equal to or higher than those in serum [71]. However, anaerobiasis at the site of deep infection may reduce the activity of gentamicin, despite its good bone penetration [24]. Gentamicin is routinely used in combination with flucloxacillin as a prophylactic agent in both elective and trauma surgery [27]. Also, gentamicin is a key antibiotic in local therapy strategies for orthopedic trauma. Gentamicin-loaded bone cements are widely used to prevent periprosthetic infections in total joint arthroplasties [97]. The elution of gentamicin is influenced by the type of cement used; for example, the antibiotic release is more effective in Palacos than in Simplex cements [96]. Conversely, the addition of tobramycin to Simplex cement provides good activity against 98% of the clinically relevant pathogens in orthopedic infections [96]. Gentamicin-eluting PMMA beads and sponges are also used to treat open fractures, PJIs, septic arthritis, and osteomyelitis, delivering highly effective antibacterial concentrations [26,27,98]. Gentamicin-coated prostheses have been used in clinical settings for megaprostheses in large periarticular bone defects, and for fracture fixation [26]. However, the presence of biofilms or gentamicin-resistant bacteria can reduce the antibiotic’s efficacy. For instance, Cara et al. (2021) [99] showed that using bone cements loaded with gentamicin plus vancomycin or clindamycin for prosthesis fixation can help in preventing PJIs caused by gentamicin-resistant staphylococci.

The primary side effects of systemic aminoglycosides are nephrotoxicity (3–11% of patients) and ototoxicity; the incidence of these adverse reactions with topical administration remains unclear [95]. Aminoglycoside resistance occurs through several mechanisms, including enzymatic modification and the inactivation of the antibiotics, target site modification via enzymes or chromosomal mutations, and efflux pumps [94]. Aminoglycoside-modifying enzymes are classified into three major families: acetyltransferases, nucleotidyl (adenyl)-transferases, and phosphotransferases, each with numerous variants. Acetyltransferases are mainly involved in the development of resistance to amikacin and tobramycin; nucleotidyltransferases modify amikacin and gentamicin, and phosphotransferases confer resistance to all three antibiotics. This mechanism of resistance is commonly observed in both Gram-positive and Gram-negative bacteria. Efflux pump activity has been linked to resistance in *Pseudomonas aeruginosa*, *Acinetobacter baumanii*, *Escherichia coli* and *Mycobacteria* spp. [94]. A relatively new mechanism, involving acquired 16S ribosomal RNA methyltransferases in Gram-negative bacteria (such as Enterobacteriaceae), supports high-level resistance to key aminoglycosides, namely gentamicin, tobramycin, and amikacin. This poses a significant threat, as it limits therapeutic options for multidrug-resistant and extensively drug-resistant Gram-negative bacterial infections [100].

#### 3.3.6. Daptomycin

Daptomycin is a cyclic lipopeptide that acts bactericidal against Gram-positive bacteria, including MRSA and VRE. It is the second most important anti-MRSA drug. Initially approved in 2003 for the treatment of SSIs caused by *S. aureus* (including MRSA), *S. pyogenes*, *S. agalactiae*, *S. dysgalactiae* sub-spp. *equisimilis*, and *E. faecalis* (vancomycin-susceptible strains), daptomycin was dosed at 4 mg/kg per day intravenously. In 2006, its indication was expanded to include the treatment of *S. aureus* bloodstream infections (bacteremia), with a higher dose of 6 mg/kg once daily [90]. Daptomycin has proven effective against multidrug-resistant Gram-positive pathogens commonly found in osteomyelitis and orthopedic infections, including PJIs, even when first-line antibiotics have failed [90,101]. While not routinely recommended for orthopedic device-related infections, daptomycin can be considered for selected cases, especially in combination with rifampicin, with doses ranging from 6 to 10 mg/kg once daily [81]. Small studies involving heterogeneous subjects (with varying types of PJIs and surgical approaches) suggest that daptomycin (4 to 8 mg/kg/day) combined with rifampicin could be an alternative option to treat MRSA PJIs [64]. Daptomycin is also an option for vancomycin-allergic patients [36]. The daptomycin’s mechanisms of action include disruptingbacterial cell membrane functions, the loss of intracellular components (such as K^+^, Mg^2+^, and ATP), and alterating cell envelope homeostasis, resulting in bacterial cell death without causing cell wall lysis [90,102].

Although daptomycin can penetrate biofilms, it does not prevent biofilms’ formation. In vitro, it acts synergistically with aminoglycosides (e.g., gentamicin) and beta-lactams. The combination of daptomycin and oxacillin has shown a good activity against intraosteoblastic MSSA and MRSA [103]. Resistance to daptomycin has emerged in recent years, primarily due to mutations in various genes (dltABCD genes, mprF, and rpoB), which lead to changes in the membrane morphology and function of *Staphylococcus aureus* strains. However, daptomycin remains active against staphylococci and enterococci. When combined with linezolid, daptomycin has shown the best performance against CoNS, with low resistance rates (0.1–0.3%) [104]. One advantage of daptomycin is its once-daily intravenous administration, which is particularly beneficial in outpatient or community settings. However, it carries a risk of myopathies, and creatine kinase levels should be monitored weekly during treatment [62].

#### 3.3.7. Fluoroquinolones

They are a class of synthetic antibiotics with potent bactericidal activity against a wide range of clinically significant Gram-negative and Gram-positive pathogens involved in bone infections. Among the four generations, fluoroquinolones from the second and third generations are primarily used for bone and joint infections. The second-generation fluoroquinolones (norfloxacin, ciprofloxacin, lomefloxacin) are active against all Gram-negative bacteria (including *Pseudomonas* spp.), some Grame-positive bacteria (such as *Staphylococcus aureus)* and atypical organisms like *Mycoplasma pneumoniae* and *Chlamydia pneumoniae*. The third generation (sparfloxacin, grepafloxacin) offer expanded activity against Gram-positive and atypical bacteria. The fourth-generation fluoroquinolones (clinafloxacin, moxifloxacin, gatifloxacin) further extend their spectrum to include anaerobes, in addition to exhibiting markedly enhanced antimicrobial activity [105,106,107]. Fluoroquinolones exert their bactericidal activity by interfering with DNA synthesis, inhibiting DNA replication and transcription via topoisomerase II type enzymes, DNA gyrase and DNA topoisomerase IV [106].

The emergence of fluoroquinolones resistance has become a critical issue, limiting their use. These antibiotics are among the most likely to drive antimicrobial resistance in hospital settings. The mechanisms of resistance include mutations in the genes encoding the target enzymes, genomic alterations that affect antibiotic accumulation and permeation, and plasmid acquisition from resistant strains in the environment [106,107]. The DNA gyrase mutations are involved in the emergence of Gram-negative bacteria resistance, while topoisomerase IV mutations are responsible for resistance in Gram-positive bacteria [107]. Resistance can also arise from the reduced intracytoplasmic accumulation of fluoroquinolones due to the down-regulation of porin proteins and decreased membrane permeability in Gram-negative bacteria, as well as the over-expression of multidrug efflux pumps in both Gram-positive and Gram-negative pathogens [107].

Newer fluoroquinolones, such as levofloxacin, moxifloxacin, gatifloxacin, and gemifloxacin, generally have lower MICs for Gram-positive bacteria compared to older fluoroquinolones (e.g., ciprofloxacin and ofloxacin), and they are associated with a lower risk of resistance emergence [55]. Among these, moxifloxacin is particularly noted for having the lowest risk of resistance development [58]. Fluoroquinolones are useful in treating PIJs caused by susceptible pathogens. Oral ciprofloxacin has been successfully used in combination with rifampicin for staphylococcal PIJs, often through an intravenous to oral switch [108]. Levofloxacin is widely used to prevent postoperative infections due to its broad antibacterial spectrum, strong antibacterial activity, low drug resistance rate, and high tissue availability [109]. Fluoroquinolones are a first-line choice for the oral treatment of Gram-negative osteomyelitis, either alone or in combination with other agents. Their main advantages include high bioavailability, excellent bone penetration, and anti-biofilm properties [55]. The high bone availability of fluroroquinolones also makes them suitable for outpatient therapy in chronic osteomyelitis, allowing for oral switch treatment [24,55].

Newer fluoroquinolones are preferred for antistaphylococcal therapy in chronic osteomyelitis, and can be used in combination with other agents when possible. However, they may be used in monotherapy when no alternative options are available. The second-generation fluoroquinolones should not be used as a monotherapy for fracture-related staphylococcal infections due to the rapid emergence of resistance and the high rate of treatment failure [55].

Resistance can develop during therapy, mainly with fluoroquinolones, rifampicin or fusidic acid, or during anti-*Pseudomonas aeruginosa* treatment when fluoroquinolones are used. Therefore, fluoroquinolones should not be employed during the initial stage of treatment in these cases [72]. The risk of fluoroquinolone resistance is higher when there is a significant bioburden, in the early postoperative period, or when patients have drains and oozing wounds [72]. When the wound is dry and based on antibiogram results, oral fluroquinolones may be used as a monotherapy for fracture-related Enterobacteriaceae infections, as they are effective in eradicating Gram-negative rods in young biofilms. In cases of fluoroquinolone resistance, high doses of oral sulfamethoxazole/trimethoprim (800/160 mg every 8 h) or beta-lactams are indicated [58].

The prolonged use of fluoroquinolones also requires careful monitoring of patients due to the possibility of serious side effects, such as tendon rupture, tendonitis, neuropathies and other neurological reactions, and aortic aneurysms. The concomitant use of fluoroquinolones and corticosteroids should be avoided due to an increased risk of tendon rupture [110].

#### 3.3.8. Linezolid

Linezolid is a synthetic oxazolidinone from a new class of antibiotics, known for its potent activity against all Gram-positive cocci, including MRSA, VRSA, VRE (both *E. faecium* and *E. faecalis*), and obligate anaerobes [24]. It is particularly valuable for treating patients with orthopedic infections, especially chronic osteomyelitis caused by the aforementioned Gram-positive cocci [81]. Linezolid boasts excellent pharmacokinetics, with 100% oral bioavailability. In addition, the antibiotic shows good bone penetration, achieving concentrations above the MICs of most Gram-positive pathogens, including multi-resistant strains. This makes linezolid a preferred option for the oral treatment of orthopedic infections, and an effective alternative to intravenous antibiotics in osteomyelitis treatment [81]. In combination with rifampicin, linezolid exhibits superior anti-biofilm activity compared to monotherapy in staphylococcal infections [81]. As well as tigecycline, a glycylcycline antibiotic, linezolid is considered a second-line agent for infections with obligate anaerobes. Metronidazole is the first-choice drug for such infections [55].

As a bacteriostatic antibiotic, linezolid inhibits bacterial protein synthesis by interfering with translation [111]. Although bactericidal antibiotics are generally preferred for treating osteomyelitis, linezolid’s excellent pharmacokinetic properties can counterbalance this disadvantage [112]. However, long-term use (beyond the recommended 28-day duration) may lead to side effects such as peripheral and optic neuropathy, as well as bone marrow suppression, primarily in patients treated for extended periods [55,90].

## 4. Conclusions

Antimicrobials play a crucial role in the prevention and treatment of orthopedic infections. Understanding the activity, safety, and tolerability of antibiotics (both individually and in combination) is essential for shaping the optimal antimicrobial management of bone and joint infections in clinical practice. Antimicrobial resistance imposes a significant global challenge in orthopedics, affecting prevention, management, and outcomes. Addressing antimicrobial resistance in orthopedics requires a multifaceted approach. The prudent use of antibiotics, including appropriate dosage, combination therapies, and treatment duration, is key to minimizing the emergence of multidrug-resistant pathogens. In addition, strict prevention protocols and robust control measures across healthcare settings are essential to limit the spread of resistant microorganisms. Implementing stringent antibiotic stewardship programs in orthopedics, along with ongoing research into new antimicrobial agents and alternative therapeutic strategies, are crucial steps in overcoming antimicrobial resistance [113,114]. Global collaboration is vital for monitoring resistance patterns and enforcing policies that reduce antibiotic misuse. The threat posed by antimicrobial resistance in orthopedics emphasizes the urgent need for coordinated, immediate action to preserve the effectiveness of antibiotics, ensure successful surgical outcomes, and protect public health worldwide.

## Data Availability

All data are available in the article and in the references.

## Supplementary Materials

The following supporting information can be downloaded at: https://www.mdpi.com/article/10.3390/medicina60121988/s1, Table S1: Biomarkers for the assessment of postoperative complications in orthopedics; Table S2: Main antimicrobials in the orthopedic infections.

## References

[1] Thakore R.V., Greenberg S.E., Shi H., Foxx A.M., Francois E.L., Prablek M.A., Nwosu S.K., Archer K.R., Ehrenfeld J.M., Obremskey W.T. (2015). Surgical site infection in orthopedic trauma: A case-control study evaluating risk factors and cost. J. Clin. Orthop. Trauma.

[2] Szymski D., Walter N., Hierl K., Rupp M., Alt V. (2024). Direct hospital costs per case of periprosthetic hip and knee joint infections in Europe—A systematic review. J. Arthroplast..

[3] Cook G.E., Markel D.C., Ren W., Webb L.X., McKee M.D., Schemitsch E.H. (2015). Infection in orthopaedics. J. Orthop. Trauma.

[4] Sanders F.R.K., Goslings J.C., Mathôt R.A.A., Schepers T. (2019). Target site antibiotic concentrations in orthopedic/trauma extremity surgery: Is prophylactic cefazolin adequately dosed? A systematic review and meta-analysis. Acta Orthop..

[5] MacLawhorn A.S., Nawabi D.H., Ranawat A.S. (2016). Management of resistant, atypical and culture-negative periprosthetic joint infections after hip and knee arthroplasty. Open Orthop. J..

[6] Li T., Zhang H., Chan P.K., Fung W.C., Fu H., Chiu K.Y. (2022). Risk factors associated with surgical site infections following joint replacement surgery: A narrative review. Arthroplasty.

[7] Mulita F., Liolis E., Akinosoglou K., Tchabashvili L., Maroulis I., Kaplanis C., Vailas M., Panos G. (2022). Postoperative sepsis after colorectal surgery: A prospective single-center observational study and review of the literature. Prz. Gastroenterol..

[8] Lakomkin N., Sathiyakumar V., Wick B., Shen M.S., Jahangir A.A., Mir H., Obremskey W.T., Dodd A.C., Sethi M.K. (2017). Incidence and predictive risk factors of postoperative sepsis in orthopedic trauma patients. J. Orthop. Traumatol..

[9] Triantafyllopoulos G., Stundner O., Memtsoudis S., Poultsides L.A. (2015). Patient surgery, and hospital related risk factors for surgical site infections following total hip arthroplasty. Sci. World J..

[10] Gonzalez C.A., O’Mara A., Cruz J.P., Roth D., Van Rysselberghe N.L., Gardner M.J. (2023). Postoperative sepsis and septic shock after hip fracture surgery. Injury.

[11] Verras G.I., Mulita F. (2024). Butyrylcholinesterase levels correlate with surgical site infection risk and severity after colorectal surgery: A prospective single-center study. Front. Surg..

[12] Javed H., Olanrewaju O.A., Ansah O.F., Ansah Owusu F., Saleem A., Pavani P., Tariq H., Vasquez Ortiz B.S., Ram R., Varrasi G. (2023). Challenges and solutions in postoperative complications: A narrative review in general surgery. Cureus.

[13] Laishram K., Borgohain B., Laishram A., Khonglah T.G., Rurar A.A., Debbarma S. (2024). Serum IL-6 as a surrogate biomarker of post-operative complications in invasive orthopaedic surgeries: A prospective observational study. Indian J. Orthop..

[14] Liu J.-J., Yang M.-S., Cen C.-Q., Xu C., Yuan H. (2018). Surgical stress biomarkers as early predictors of postoperative infectious complications in patients with colorectal cancer. Transl. Cancer Res..

[15] Amanai E., Nakai K., Saito J., Hashiba E., Miura T., Morohashi H., Sakamoto Y., Mikami A., Hakamada K., Hirota K. (2022). Usefulness of presepsin for the early detection of infectious complications after elective colorectal surgery, compared with C-reactive protein and procalcitonin. Sci. Rep..

[16] Vallet H., Chenevier-Gobeaux C., Villain C., Cohen-Bittan J., Ray P., Epelboin L., Vernu M., Riou B., Khiami F., Boddaert J. (2017). Prognostic value of serum procalcitonin after orthopedic surgery in the elderly population. J. Gerontol. A.

[17] Goulart A., Ferreira C., Estrada A., Nogueira F., Martins S., Mesuita-Rodrigues A., Sousa N., Leão P. (2018). Early inflammatory biomarkers as predictive factors for freedom from infection after colorectal cancer surgery. Surg. Infect..

[18] Herman T.F., Popowicz P., Bordoni B. (2024). Wound Classification. StatPearls.

[19] Benjamin W., Sessums P., Shi G., Libertin C., Heckman M., Brushaber D., Ledfort C. (2022). An alternative wound class for orthopedic surgery is more strongly associated with risk for postoperative infection in total joint arthroplasty than the current Surgical Wound Classification System. J. Orthop. Exp. Innov..

[20] Onyekwelu I., Yakkanti R., Protzer L., Pinkston C.M., Tucker C., Seligson D. (2017). Surgical wound classification and surgical site infections in the orthopaedic patient. J. Am. Acad. Orthop. Surg. Glob. Res. Rev..

[21] Salam M.A., Al-Amin M.Y., Salam M.T., Pawar J.S., Akhter N., Rabaan A.A., Alqumber M.A.A. (2023). Antimicrobial resistance: A growing serious threat for global public health. Healthcare.

[22] World Health Organization Global Antimicrobial Resistance and Use Surveillance System (GLASS) Report 2021. https://iris.who.int/bitstream/handle/10665/341666/9789240027336-eng.pdf?sequence=1.

[23] Elifranji Z.O., Haddad B., Salameh A., Alzubaidi S., Yousef N., Al Nawaiseh M., Alkhatib A., Aburumman R., Karam A.M., Azzam M.I. (2022). Microbiological profile and drug resistance analysis of postoperative infections following orthopedic surgery: A 5-year retrospective review. Adv. Orthop..

[24] Darley E.S.R., MacGowan A.P. (2004). Antibiotic treatment of Gram-positive bone and joint infections. J. Antimicrobial. Chemother..

[25] Papadopoulos A., Ribera A., Mavrogenis A.F., Rodriguez-Pardo D., Bonnet E., Salles M.J., del Toro M.D., Nguyen S., Blanco-Garcia A., Skaliczki G. (2019). Multidrug-resistant and extensively drug-resistant Gram-negative prosthetic joint infections: Role of surgery and impact on colistin administration. Int. J. Antimicrob. Agents.

[26] Masters E.A., Ricciardi B.F., Bentley K.L.M., Moriarty T.F., Schwarz E.M., Muthukrishnan G. (2022). Skeletal infections: Microbial pathogenesis, immunity and clinical management. Nat. Rev. Microbiol..

[27] Bryson D.J., Morris D.L.J., Shivji F.S., Rollins K.R., Snape S., Ollivere B.J. (2016). Antibiotic prophylaxis in orthopaedic surgery: Difficult decisions in an era of evolving antibiotic resistance. Bone Jt. J..

[28] Wang B., Wang Q., Hamushan M., Yu J., Jiang F., Li M., Guo G., Tang J., Han P., Shen H. (2023). Trends in microbiological epidemiology of orthopedic infections: A large retrospective study from 2008 to 2021. BMC Infect. Dis..

[29] Gatti M., Barnini S., Guarracino F., Parisio E.M., Spinicci M., Viaggi B., D’Arienzo S., Forni S., Galano A., Gemmi F. (2022). Orthopaedic implant-associated Staphylococcal infections: A critical reappraisal of unmet clinical needs associated with the implementation of the best antibiotic choice. Antibiotics.

[30] de Buys M., Moodley K., Cakic J.N., Pietrzak J.R.T. (2023). *Staphylococcus aureus* colonization and periprosthetic joint infection in patients undergoing elective total joint arthroplasty: A narrative review. EFORT Open Rev..

[31] Li B., Webster T.J. (2018). Bacteria antibiotic resistance: New challenges and opportunities for implant-associated orthopedic infecions. J. Orthop. Res..

[32] World Health Organization AMR Surveillance: Global Antimicrobial Resistance Data 2022. https://www.who.int/publications/i/item/9789240062702.

[33] Tsang S.J., Simpson A.H.R.W. (2020). Antimicrobial rationing in orthopaedic surgery. Bone Jt. Res.

[34] Lefko C.T., Kim B.I., Schwartz A.M., Case A., Seidelman J.L., Hendershot E.F., Bolognesi M.P., Seyler T.M., Jiranek W.A. (2021). Prosthetic knee infection with coagulase-negative *Staphylococcus*: A harbinger of poor outcomes. J. Arthroplast..

[35] Wang Y., Liu C., Xia W., Cui Y., Yu L., Zhao D., Guan X., Wang Y., Wang Y., Li Y. (2024). Association of coagulase-negative staphylococci with orthopedic infections detected by in-house multiplex real-time PCR. Front. Microbiol..

[36] European Centre for Disease Prevention and Control Antimicrobial Resistance in the EU/EEA 2022. https://www.ecdc.europa.eu/sites/default/files/documents/antimicrobial-resistance-policy-brief-2022.pdf.

[37] Boisrenoult P. (2018). Cutibacterium acnes prosthetic joint infection: Diagnosis and treatment. Orthop. Traumatol. Surg. Res..

[38] Bondarenko S., Chang C.B., Cordero-Ampuero J., Kates S., Kheir M., Klement M.R., McPherson E., Morata L., Silibovsky R., Skaliczki G. (2019). General assembly, prevention, antimicrobials (systemic): Proceedings of International Consensus on Orthopedic Infections. J. Arthroplast..

[39] Theuretzbacher U. (2017). Global antimicrobial resistance in Gram-negative pathogens and clinical need. Curr. Opin. Microbiol..

[40] World Health Organization Guidelines for the Prevention and Control of Carbapenem-Resistant Enterobacteriaceae, *Acinetobacter baumanii* and *Pseudomonas aeruginosa* in Health Care Facilities 2017. https://iris.who.int/bitstream/handle/10665/259462/9789241550178-eng.pdf;jsessionid=D086CBA35BEEC6590A5CB0B52988238D?sequence=1.

[41] Rodríguez-Pardo D. (2019). Antibiotic prophylaxis in orthopaedic surgery: Clinical practice guidelines or individualized prophylaxis?. Enferm. Infecc. Microbiol. Clin..

[42] Bianco Prevot L., Tansini L., Riccardo A., Bolcato V., Tronconi L.P., Basile G. (2024). Cutting periprosthetic infection rate: *Staphylococcus aureus* decolonization as a mandatory procedure in preoperative knee and hip replacement care-insights from a systematic review and meta-analysis of more than 50,000 patients. J. Clin. Med..

[43] Portais A., Gallouche M., Pavese P., Caspar Y., Bosson J.-L., Astagneau P., Pailhé R., Tonetti J., Duval B.R., Landelle C. (2024). *Staphylococcus aureus* screening and preoperative decolonisation with Mupirocin and Chlorhexidine to reduce the risk of surgical site infections in orthopaedic surgery: A pre-post study. Antimicrob. Resist. Infect Control.

[44] Zhu X., Sun X., Zeng Y., Feng W., Li J., Zeng J., Zeng Y. (2020). Can nasal *Staphylococcus aureus* screening and decolonization prior to elective total joint arthroplasty reduce surgical site and prosthesis-related infections? A systematic review and meta-analysis. J. Orthop. Surg..

[45] Young B.C., Votintseva A.A., Foster D., Godwin H., Miller R.R., Anson L.W., Walker A.S., Peto T.E.A., Crook D.W., Knox K. (2017). Multi-site and nasal swabbing for carriage of *Staphylococcus aureus*: What does a single nose swab predict?. J. Hosp. Infect..

[46] Righi E., Mutter N.T., Guirao X., Del Toro M.D., Eckmann C., Friedrich A.W., Giannella M., Presterl E., Christaki E., Cross L.A. (2024). European Society of Clinical Microbiology and Infectious Diseases/European Committee on infection control clinical guidelines on pre-operative decolonization and targeted prophylaxis in patients colonized by multidrug-resistant Grampositive bacteria before surgery. Clin. Microbiol. Infect..

[47] Erwin D.Z., Chen P. (2024). Mupirocin. StatPearls.

[48] AlBuhairan B., Hind D., Hutchinson A. (2008). Antibiotic prophylaxis for wound infections in total joint arthroplasty: A systematic review. J. Bone Jt. Surg. Br..

[49] Dhammi I.K., Haq R.U., Kumar S. (2015). Prophylactic antibiotics in orthopedic surgery. Indian J. Orthop..

[50] European Centre for Disease Prevention and Control Systematic Review and Evidence-Based Guidance on Perioperative Antibiotic Prophylaxis 2013. https://www.ecdc.europa.eu/sites/default/files/media/en/publications/Publications/Perioperative%20antibiotic%20prophylaxis%20-%20June%202013.pdf.

[51] Upadhyyaya G.K., Tewari S. (2017). Enhancing surgical outcomes: A critical review of antibiotic prophylaxis in orthopedic surgery. Cureus.

[52] Wuarin L., Abbas M., Harbarth S., Waibel F., Holy D., Burkhard J., Uçkay I. (2019). Changing perioperative prophylaxis during antibiotic therapy and iterative debridement for orthopedic infections?. PLoS ONE.

[53] Bernard L., Hoffmeyer P., Assal M., Vaudaux P., Schrenzel J., Lew D. (2004). Trends in the treatment of orthopaedic prosthetic infections. J. Antimicrob. Chemother..

[54] Richards G.A., Brink A.J., Feldman C. (2019). Rational use of the fluoroquinolones. SAMJ.

[55] Fantoni M., Taccari F., Giovannenze F. (2019). Systemic antibiotic treatment of chronic osteomyelitis in adults. Eur. Rev. Med. Pharmacol. Sci..

[56] Koch B.C.P., Zhao Q., Oosterhoff M., van Oldenrijk J., Abdulla A., de Winter B.C.M., Bos K., Muller A.E. (2022). The mysteries of target site concentrations of antibiotics in bone and joint infections: What is known? A narrative review. Expert Opin. Drug Metab. Toxicol..

[57] Zimmerli W., Moser C. (2012). Pathogenesis and treatment concepts of orthopaedic biofilm infections. FEMS Immunol. Med. Microbiol..

[58] Depypere M., Kuehl R., Metsemakers W.-J., Senneville E., McNally M.A., Obremskey W.T., Zimmerli W., Atkins B.L., Trampuz A. (2020). Recomandations for systemic antimicrobial therapy in fracture-related infection: A consensus from an International Expert Group. J. Orthop. Trauma.

[59] Metsemakers W.-J., Fragomen A.T., Moriarty F., Morgenstern M., Egol K.A., Zalavras C., Obremskey W.T., Raschke M., McNally M.A. (2020). Evidence-based recommendations for local antimicrobial strategies and dead space management in fracture-related infection. J. Orthop. Trauma.

[60] Flores M.J., Brown K.E., Morshed S., Shearer D.W. (2022). Evidence for local antibiotics in the prevention of infection in orthopaedic trauma. J. Clin. Med..

[61] Chang Y., Tai C.L., Hsieh P.H., Ueng S.W. (2013). Gentamicin in bone cement: A potentially more effective prophylactic measure of infectionin joint arthroplasty. Bone Jt. Res..

[62] Winner J.S., Murphy C.V. (2010). Bone and Joint Infections. PSAP Seventh Edition, Infectious Diseases.

[63] MSD Manual Professional Version 2024. https://www.msdmanuals.com/professional/infectious-diseases/bacteria-and-antibacterial-medications.

[64] Le Vavasseur B., Zeller V. (2022). Antibiotic therapy for prosthetic joint infections: An overview. Antibiotics.

[65] Raj G.M., Abialbon P., Nishanthi A., Jayanthi M., Marshall Raj G. (2021). Penicillins, Cephalosporins and Other Beta-Lactam Antibiotics. Introduction to Basics of Pharmacology and Toxicology, Vol. 2: Essentials of Systemic Pharmacology: From Principles to Practice.

[66] Bui T., Patel P., Preuss C.V. (2024). Cephalosporins. StatPearls.

[67] Armstrong T., Fenn S.J., Hardie K.R. (2021). JMM Profile: Carbapenems: A broad-spectrum antibiotic. J. Med. Microbiol..

[68] Hawkey P.M., Livermore D.M. (2012). Carbapenem antibiotics for serious infections. BMJ.

[69] Pandey N., Cascella M. (2024). Beta-lactam Antibiotics. StatPearls.

[70] Yeap J.S., Lim J.W., Vergis M., Au Yeung P.S., Chiu C.K., Singh H. (2006). Prophylactic antibiotics in orthopaedic surgery: Guidelines and practice. Med. J. Malays..

[71] Thabit A.K., Fatani D.F., Bamakhrama M.S., Barnawi O.A., Basudan L.O., Alheaili S.F. (2019). Antibiotic penetration into bone and joints: An updated review. IJID.

[72] The Korean Society for Chemotherapy, The Korean Society of Infectious Diseases, The Korean Orthopaedic Association (2014). Clinical guidelines for the antimicrobial treatment of bone and joint infections in Korea. Infect. Chemother..

[73] Khorrami M., Tabatabaei S., Ahmadarabi M. (2012). The efficacy of short-term vs. long-term antibiotic therapy in preventing deep infection after orthopedic procedures (a prospective observational study). Jundishapur. J. Microbiol..

[74] Zawadsky M.W., Scherping S.C., Wiesel S.W., Delahay J.N. (2010). Orthopedic infections. Essentials of Orthopedic Surgery.

[75] Aminov R. (2017). History of antimicrobial drug discovery: Major classes and health impact. Biochem. Pharmacol..

[76] Saunders W.B., Constable P.D., Hinchcliff K.W., Done S.H., Grünberg W. (2017). Practical Antimicrobial Therapeutics. Veterinary Medicine.

[77] Renz N., Trampuz A., Zimmerli W. (2021). Controversy about the role of rifampin in biofilm infections: Is it justified?. Antibiotics.

[78] Jacqueline C., Caillon J. (2014). Impact of bacterial biofilm on the treatment of prosthetic joint infections. J. Antimicrob. Chemother..

[79] Zimmerli W., Sendi P. (2019). Role of rifampin against Staphylococcal biofilm infections in vitro, in animal models, and in orthopedic-device-related infections. Antimicrob. Agents Chemother..

[80] Sendi P., Zimmerli W. (2017). The use of rifampin in staphylococcal orthopaedic-device-related infections. Clin. Microbiol. Infect..

[81] Sendi P., Zimmerli W. (2012). Antimicrobial treatment concepts for orthopaedic device-related infection. Clin. Microbiol. Infect..

[82] Fernandes P. (2016). Fusidic Acid: A bacterial elongation factor inhibitor for the oral treatment of acute and chronic Staphylococcal infections. Cold Spring Harb. Perspect. Med..

[83] Murphy P.B., Bistas K.G., Patel P., Le J.K. (2024). Clindamycin. StatPearls.

[84] Álvarez A.L., Van de Sijpe G., Desmet S., Metsemakers W.-J., Spriet I., Allegaert K., Rozenski J. (2022). Ways to improve insights into clindamycin pharmacology and pharmacokinetics tailored practice. Antibiotics.

[85] Bonnaire A., Vernet-Garnier V., Lebrun D., Bajolet O., Bonnet M., Hentzien M., Ohl X., Dialli S., Bani-Sadr F. (2021). Clindamycin combination treatment for the treatment of bone and joint infections caused by clindamycin-susceptible, erythromycin-resistant *Staphylococcus* spp. Diagn. Microbiol. Infect. Dis..

[86] Kang H.-K., Park Y. (2015). Glycopeptide antibiotics: Structure and mechanisms of action. J. Bacteriol. Virol..

[87] Binda E., Marinelli F., Marcone G.L. (2014). Old and new glycopeptide antibiotics: Action and resistance. Antibiotics.

[88] Bruniera F.R., Ferreita F.M., Saviolli L.R.M., Bacci M.R., Feder D., Gonçalves Pedreira D.L., Sorgini Peterlini M.A., Azzalis L.A., Campos Junueira V.B., Fonseca F.L.A. (2015). The use of vancomycin with its therapeutic and adverse effects: A review. Eur. Rev. Med. Pharmacol. Sci..

[89] Saleh A., Khanna A., Chagin K.M., Klika A.K., Johnston D., Barsoum W.J. (2015). Glycopeptides versus β-lactams for the prevention of surgical site infections in cardiovascular and orthopedic surgery: A meta-analysis. Ann. Surg..

[90] Rice D.A.K., Mendez-Vigo L. (2009). Daptomycin in bone and joint infections: A review of the literature. Arch. Orthop. Trauma Surg..

[91] Soriano A., Popescu D., García S., Bori G., Martínez J.A., Balasso V., Marco F., Almela M., Mensa J. (2006). Usefulness of teicoplanin for preventing methicillin-resistant *Staphylococcus aureus* infections in orthopedic surgery. Eur. J. Clin. Microbiol. Infect. Dis..

[92] Capdevila A., Navarro M., Bori G., Tornero E., Camacho P., Bosch J., Garcia S., Mensa J., Soriano A. (2016). Incidence and risk factors for infection when teicoplanin is included for prophylaxis in patients with hip fracture. Surgery.

[93] Van Bambeke F. (2015). Lipoglycopeptide antibacterial agents in Gram-Positive infections: A comparative review. Drugs.

[94] Krause K.M., Serio A.W., Kane T.R., Connolly L.E. (2016). Aminoglycosides: An overview. Cold Spring Harb. Perspect. Med..

[95] Thy M., Timsit J.F., de Montmollin E. (2023). Aminoglycosides for the Treatment of Severe Infection Due to Resistant Gram-Negative Pathogens. Antibiotics.

[96] Bistolfi A., Massazza G., Verné E., Massè A., Deledda D., Ferraris S., Miola M., Galetto F., Crova M. (2011). Antibiotic-loaded cement in orthopedic surgery: A review. ISRN Orthop..

[97] Thomas B., Benedikt M., Alamri A., Kapp F., Bader R., Summer B., Thomas P., Oppel E. (2020). The role of antibiotic-loaded bone cement in complicated knee arthroplasty: Relevance of gentamicin allergy and benefit from revision surgery-a case control follow-up study and algorithmic approach. J. Orthop. Surg. Res..

[98] Barth R.E., Vogely H.C., Hoepelman A.I., Peters E.J. (2011). To bead or not to bead? Treatment of osteomyelitis and prosthetic joint-associated infections with gentamicin bead chains. Int. J. Antimicrob. Agents.

[99] Cara A., Ballet M., Hemery C., Ferry T., Laurent F., Josse J. (2021). Antibiotics in bone cements used for prosthesis fixation: An efficient way to prevent *Staphylococcus aureus* and *Staphylococcus epidermidis* prosthetic joint infection. Front. Med..

[100] Doi Y., Wachino J.I., Arakawa Y. (2016). Aminoglycoside Resistance: The Emergence of acquired 16S ribosomal RNA methyltransferases. Infect. Dis. Clin. North Am..

[101] Shiels S.M., Tennent D.J., Wenke J.C. (2018). Topical rifampin powder for orthopedic trauma part I: Rifampin powder reduces recalcitrant infections in a delayed treatment musculoskeletal trauma model. J. Orthop. Res..

[102] Heidary M., Khosravi A.D., Khoshnood S., Nasiri M.J., Soleimani S., Goudarzi M. (2018). Daptomycin. J. Antimicrobial. Chemother..

[103] Telles J.P., Cieslinski J., Tuon F.F. (2019). Daptomycin to bone and joint infections and prosthesis joint infections: A systematic review. Braz. J. Infect. Dis..

[104] Shariati A., Dadashi M., Chegini Z., van Belkum A., Mirzaii M., Khoramrooz S.S., Darban-Sarokhalil D. (2020). The global prevalence of daptomycin, tigecycline, quinupristin/dalfopristin, and linezolid-resistant *Staphylococcus aureus* and coagulase–negative staphylococci strains: A systematic review and meta-analysis. Antimicrob. Resist. Infect. Control.

[105] Sharma P.C., Jain A., Jain S. (2009). Fluoroquinolone antibacterials: A review on chemistry, microbiology and therapeutic prospects. Acta Pol. Pharm. Drug Res..

[106] Pham T.D.M., Ziora Z.M., Blaskovich M.A.T. (2019). Quinolone antibiotics. Medchemcomm.

[107] Lungu I.-A., Moldovan O.-L., Biriș V., Rusu A. (2022). Fluoroquinolones hybrid molecules as promising antibacterial agents in the fight against antibacterial resistance. Pharmaceutics.

[108] Berdal J.E., Skråmm I., Mowinckel P., Gulbrandsen P., Bjørnholt J.V. (2005). Use of rifampicin and ciprofloxacin combination therapy after surgical debridement in the treatment of early manifestation prosthetic joint infections. Clin. Microbiol. Infect..

[109] Wang W., Zhou C.Q., Zhang J. (2022). Clinical efficacy and safety analysis of levofloxacin for the prevention of infection after traumatic osteoarthrosis and internal fixation: Systematic review and meta-analysis. Emerg. Med. Int..

[110] Rusu A., Munteanu A.-C., Arbănași E.-M., Uivarosi V. (2023). Overview of side-effects of antibacterial fluoroquinolones: New drugs versus old drugs, a Step forward in the safety profile?. Pharmaceutics.

[111] Azzouz A., Preuss C.V. (2024). Linezolid. StatPearls.

[112] Theil C., Schmidt-Braekling T., Gosheger G., Schwarze J., Dieckmann R., Schneider K.N., Möllenbeck B. (2020). Clinical use of linezolid in periprosthetic joint infections—A systematic review. J. Bone Jt. Infect..

[113] Lakhani A., Jindal K., Katri K. (2024). Antimicrobial resistance (AMR) in orthopaedic surgeries: A complex issue and global threat. J. Orthop. Rep..

[114] Sirwan K.A., Safin H., Karzan Q., Radwan H.I., Abdulmalik F., Kochr A.M., Mona Gamal M. (2024). Antimicrobial resistance: Impacts, challenges, and future prospectes. J. Med. Surg. Public Health.

