# Supplementary material for: Antimicrobials in Orthopedic Infections: Overview of Clinical Perspective and Microbial Resistance
Source: Medicina (Kaunas). 2024 Dec 2;60(12):1988. doi: 10.3390/medicina60121988 (PMC11728363; doi:10.3390/medicina60121988)
Supplement: Supplementary file 1 [file medicina-60-01988-s001.zip › medicina-3308741-supplementary.pdf]

Table S1. Biomarkers for the assessment of postoperative complications in orthopedics.

Biomarker	Relevance	Reference
Butyrylcholinesterase	Low-grade systemic inflammation (but not sepsis)	[11]
C-reactive protein (CRP)	Inflammation<br>Infection	[12]
D-dimer	Thromboembolic events	[12]
Interleukin-6 (IL-6)	Monitoring for early detection of complications in the invasive orthopedic surgery	[13]
Interleukin-10 (IL-10)	Shifts the immune reaction from Th1 type to Th2 type	[14]
Lactate	Sepsis<br>Insufficient oxygen supply	[12]
Presepsin	Sepsis	[15]
Procalcitonin (PCT)	Bacterial infection<br>Sepsis	[12,16]
Serum albumin	Infection<br>Inflammation	[17]
Troponin	Damage to heart muscle, Cardiac issues	[12]
Tumor necrosis factor-α (TNF-α)	Surgical stress<br>Inflammation	[14]
White blood cell count	Infection<br>Inflammation	[17]

Table S2. Main antimicrobials in the orthopedic infections [62-64].

Antimicrobial agent	Susceptible pathogens	Dosage
Flucloxacillin	Staphylococcus aureus (MSSA), Streptococcus pyogenes	2g every 6 hours/day, iv
Nafcillin	Staphylococcus spp, Streptococcus spp	2 g every 4 hours, iv
Amoxicillin	Gram-positive: Streptococcus spp., Enterococcus spp., Listeria monocytogenes<br>Gram-negative: Haemophilus influenzae, Escherichia coli, Proteus mirabilis, Salmonella spp., Shigella spp.	1 g, 6-8 hourly/day, po
Ampicillin/sulbactam	Gram-positive: MSSA, coagulase negative Staphylococci, Streptococcus pneumoniae<br>Gram-negative: Haemophilus influenzae, Escherichia coli, Acinetobacter, Klebsiella, Neisseria meningitides, Moraxella catarrhalis<br>Anaerobes	3 g, 8 hourly/day, iv
Cefazolin	Gram-positive: MSSA, coagulase negative Staphylococci, Streptococcus pneumoniae<br>Gram-negative: Moraxella catarrhalis, Escherichia coli, Klebsiella pneumoniae, Proteus mirabilis	2 g every 8 hours/day, iv
Cefepime	Gram-positive: MSSA, coagulase negative Staphylococci, Streptococcus pneumoniae<br>Gram-negative: Haemophilus influenzae, Moraxella catarrhalis, Neisseria meningitides, Neisseria gonorrhoeae, Escherichia coli, Pseudomonas aeruginosa	2 g every 8-12 hours/day, iv
Ceftazidime	Gram-negative: Haemophilus influenzae, Moraxella catarrhalis, Neisseria meningitides, N. gonorrhoeae, Escherichia coli, Pseudomonas aeruginosa	2 g every 8-12 hours/day, iv
Ceftriaxone	Gram-positive: MSSA, Coagulase negative Staphylococci, Streptococcus	2g/day, iv

	pneumoniae (penicillin susceptible), Streptococcus spp. Gram-negative: Haemophilus influenzae, Moraxella catarrhalis, Neisseria meningitides, N. gonorrhoeae, Enterobacteriaceae, Escherichia coli	
Piperacillin/tazobactam	Gram-positive: MSSA, Coagulase negative Staphylococci, Streptococcus pneumoniae (penicillin susceptible), Streptococcus spp. Gram-negative: Haemophilus influenzae, Moraxella catarrhalis, Neisseria meningitides, N. gonorrhoeae, Enterobacteriaceae, Escherichia coli, Pseudomonas aeruginosa	4,5 g every 6-8 hours/day, iv
Ciprofloxacin	Gram-positive: MSSA, Streptococcus pneumoniae Gram-negative: Enterobacteriaceae, Haemophilus spp., Neisseria gonorrhoeae, N. meningitides, Moraxella catarrhalis, Pseudomonas aeruginosa, Stenotrophomonas maltophilia Atypicals: Legionella pneumophilia	400 mg every 8-12 hours/day, iv; 750 mg x 2/day, po
Levofloxacin	Gram-positive: MSSA, MRSA, Streptococcus pneumoniae, Listeria monocytogenes Gram- negative: Enterobacteriaceae, Haemophilus spp., Neisseria gonorrhoeae, N. meningitides, Moraxella catarrhalis, Pseudomonas aeruginosa, Stenotrophomonas maltophilia, Atypicals: Legionella pneumophilia	750 mg/day or 500 mg x 2/day, po
Moxifloxacin	Gram-positive: MSSA, MRSA, Streptococcus pneumoniae, Listeria monocytogenes Gram- negative: Enterobacteriaceae, Haemophilus spp., Neisseria gonorrhoeae, N. meningitides, Moraxella catarrhalis, Pseudomonas	400 mg/day, po/iv

	aeruginosa, Stenotrophomonas maltophilia<br>Atypicals: Legionella pneumophilia, Chlamydia pneumoniae, Mycoplasma pneumoniae	
Clindamycin	Gram-positive: Streptococcus spp., Staphylococcus spp.,<br>Gram-negative: Haemophilus influenzae, Moraxella catarrhalis, Bacteroides spp.,<br>Anaerobes: Clostridia spp., Prevotella spp., Fusobacterium spp., Veillonella spp.	600 mg every 8 hours/day, iv
Daptomycin	MSSA/MRSA and vancomycin-resistant Enterococci (VRE)	8-10 mg/kg/day, iv
Ertapenem	Gram-positive: MSSA, Streptococcus pyogenes, S. agalactiae<br>Gram-negative: Escherichia coli, Haemophilus influenzae, Moraxella catarrhalis, Klebsiella pneumoniae<br>Anaerobes: Bacteroides spp., Clostridium clostridioforme, Peptostreptococcus spp.	1g/day, iv
Meropenem	Gram-positive: Staphylococcus aureus including penicillinase-producing strains, Enterococcus spp., Streptococcus pneumoniae, S. pyogenes, S. viridans<br>Gram-negative: Acinetobacter spp., Citrobacter spp., Enterobacter cloacae, Escherichia coli, Haemophilus influenzae, Klebsiella pneumoniae, Pseudomonas aeruginosa<br>Anaerobes: Peptostreptococcus spp., Bacteroides spp., Fusobacterium spp.	1-2 g every 8 hours/day, iv
Linezolid	Gram-positive: MSSA, MRSA, methicillin-resistant Staphylococcus epidermidis (MRSE), Streptococcus pneumoniae including multidrug-resistant strains, S. pyogenes, S. agalactiae, vancomycin resistant Enterococcus faecium and faecalis	600 mgx2/day, iv/po

	(VREF)	
Gentamicin	Gram-negative: Pseudomonas aeruginosa, Escherichia coli, Proteus spp., Klebsiella spp., Enterobacter spp., Serratia spp., Providencia spp., Acinetobacter spp., Citrobacter spp., Morganella spp., Gram-positive: Staphylococcus spp., Streptococcus viridans, Enterococcus spp.	3 mg/kg, once a day, iv
Tobramycin	Gram-negative: Serratia spp., Proteus spp., Pseudomonas aeruginosa, Citrobacter, Aeromonas hydrophilia, Klebsiella spp., Escherichia coli Gram-positive: Staphylococcus spp., Streptococcus spp.	5 mg/kg, once a day, iv
Amikacin	Gram-negative: Pseudomonas aeruginosa, Escherichia coli, Proteus spp., Klebsiella spp., Enterobacter spp., Serratia spp., Providencia spp., Acinetobacter spp., Citrobacter spp., Morganella spp. Gram-positive: Staphylococcus spp., Streptococcus viridans, Enterococcus spp.	15 mg/kg, once a day, iv
Vancomycin	Staphylococcus aureus (vancomycin susceptible), Coagulase negative Staphylococci, Streptococcus spp., Enterococcus spp. (Vancomycin-susceptible), Corynebacterium jeikeium, Clostridium spp., Listeria monocytogenes, Actinomyces	15-20 mg/kg every 12 hours/day, iv
Teicoplanin	Staphylococcus aureus (teicoplanin susceptible), Coagulase negative Staphylococci, Streptococcus pneumoniae, Streptococcus spp., Enterococcus spp. (Teicoplanin susceptible), Corynebacterium jeikeium, Clostridium spp., Listeria monocytogenes, Actinomyces	12 mg/kg/day, iv
Rifampicin	Gram-positive: Staphylococcus spp., Streptococcus spp.	600 mg/day; 300-450 mg x 2/day po

	Gram-negative: Legionella spp., Bacteroides fragilis, Yersinia pestis, Coxiella burnetti, Neisseria meningitides	
Fusidic acid	Gram-positive: MSSA, MRSA, Corynebacterium spp.<br>Gram-negative: Neisseria spp, Bordetella pertussis<br>Anaerobes: Clostridium difficile, C. perfringens, Peptostreptococcus spp, Propionibacterium acnes	500 mgx 3/day, po
Metronidazole	Anaerobic Gram-negative: Bacteroides fragilis, Bacteroides species, Fusobacterium spp., Porphyromonas spp., Prevotella spp.<br>Anaerobic Gram-positive: Clostridium spp.<br>Peptostreptococcus spp., Veillonella spp.	400-500 mg,<br>8 hourly/day, po

iv, intravenous; po, *per os*
